# PD232 A New Process For Patients And Caregivers To Suggest Health Technologies For Funding Consideration In Singapore

**DOI:** 10.1017/S0266462324004513

**Published:** 2025-01-07

**Authors:** Jen Hun Koh, Fiona Pearce, Shawn Quek, Ping-Tee Tan

## Abstract

**Introduction:**

In Singapore, health technologies were previously identified for funding consideration through horizon scanning or annual applications from clinicians, before being prioritized by the Ministry of Health advisory committees for evaluation by the Agency for Care Effectiveness (ACE). This poster describes a new process co-developed with local patient organizations to enable patients and caregivers to suggest health technologies for evaluation.

**Methods:**

An application form was developed in plain language for patients and caregivers which requested the name of the health technology and its formulation, the medical condition it is used for, the perceived benefits and disadvantages, and the reasons why it should be funded. A factsheet explaining the selection process was codeveloped with patients, published online, and sent electronically with the application form in October 2023 to all local patient organizations, alongside the open application call for clinicians. Applications were accepted until January 2024 and then collated for prioritization in line with predefined selection criteria. All patients were notified of the outcome of their application.

**Results:**

Fifty applications were received from patients during the first two months of the open call compared to 75 from clinicians. Most of the patient applications (66%) requested drugs for treating asthma or respiratory conditions. Drugs requested by clinicians generally differed from those requested by patients except for 5 topics, suggesting that patients may perceive clinical need differently. Most patient applicants had used the requested drugs before and considered they were effective and convenient, but unaffordable. Health technologies were more likely to be prioritized for evaluation when their benefits were plausible and supported by evidence, and they could fill an unmet clinical need for patients.

**Conclusions:**

The process will be updated in line with feedback to encourage continued patient participation annually. Enabling patients and caregivers to suggest health technologies for evaluation provides ACE with a better understanding of their needs, preferences, lived experiences, and expectations, and ensures that subsequent funding recommendations informed by ACE’s evaluations address therapeutic gaps and improve treatment affordability and patient outcomes.

